# Increasing Awareness and Use of Mobile Health Technology Among Individuals With Hypertension in a Rural Community of Bangladesh: Protocol for a Randomized Controlled Trial

**DOI:** 10.2196/15523

**Published:** 2020-08-17

**Authors:** Yasmin Jahan, Michiko Moriyama, Md Moshiur Rahman, Kana Kazawa, Atiqur Rahman, Abu Sadat Mohammad Sayeem Bin Shahid, Sumon Kumar Das, ASG Faruque, Mohammod Jobayer Chisti

**Affiliations:** 1 Graduate School of Biomedical and Health Sciences, Hiroshima University Hiroshima Japan; 2 Department of Culture and Society, Linköping University Norrköping Sweden; 3 International Centre for Diarrheal Disease Research Dhaka Bangladesh; 4 Menzies School of Health Research Darwin, Northern Teritorry Australia

**Keywords:** mHealth, hypertension, behavioral changes, knowledge, awareness development, Bangladesh

## Abstract

**Background:**

Hypertension remains one of the foremost noncommunicable diseases that most often lead to cardiovascular diseases and its different complications. The prevalence of hypertension in Bangladesh has been increasing. However, there are very limited studies that have evaluated the impact of health education and awareness development in mitigating the burden of hypertension and its complications in Bangladesh.

**Objective:**

This study aims to increase awareness, enhance knowledge, and change lifestyle behaviors through health education and the use of mobile health (mHealth) technology among individuals with hypertension living in a rural community of Bangladesh.

**Methods:**

A randomized controlled trial is underway in a Mirzapur subdistrict of Bangladesh. This trial compares two groups of individuals with hypertension: The comparison arm receives health education and the intervention arm receives health education and a periodic mobile phone–based text message intervention. The trial duration is 5 months. The primary end point is participants’ actual behavior changes brought about by increased awareness and knowledge.

**Results:**

Enrollment of participants started in August 2018, and collection of follow-up data was completed at the end of July 2019. A total of 420 participants volunteered to participate, and among them, 209 and 211 were randomly allocated to the intervention group and the control group, respectively. Among them, the ratio of males/females was 12.0/88.0 in the intervention group and 16.1/83.9 in the control group. Data cleaning and analyses have been completed and the results have been submitted for publication.

**Conclusions:**

Periodic short education using mHealth technology in addition to face-to-face health education may be an effective method for increasing awareness and knowledge about behavioral changes and maintaining healthy lifestyle behaviors.

**Trial Registration:**

Bangladesh Medical Research Council (BMRC) 06025072017; ClinicalTrials.gov NCT03614104, https://clinicaltrials.gov/ct2/show/NCT03614104; University hospital Medical Information Network (UMIN) R000033736, https://upload.umin.ac.jp/cgi-open-bin/ctr_e/ctr_his_list.cgi?recptno=R000033736

**International Registered Report Identifier (IRRID):**

DERR1-10.2196/15523

## Introduction

### Background

Hypertension is one of the most significant modifiable risk factors for cardiovascular diseases (CVDs) worldwide [1], with increasing pervasiveness in low- and middle-income countries [2]. Currently, high blood pressure (BP) has been reported to cause 7.5 million deaths, which account for approximately 12.8% of the total deaths occurring globally [3]. Of these CVD deaths, 53% are due to complications of hypertension. High BP is called the “silent killer” because often, it has no warning symptoms or signs, and many people do not know that they have it [4]. Factors attributed to the increased prevalence of hypertension include population growth; aging population; and behavioral risk factors such as smoking, low-quality diet, harmful alcohol consumption, less physical activity, and overweight or obesity [5].

A review of community-based interventions for CVD implemented in low- and middle-income countries recommends that patient education can have a positive effect on treatment adherence and BP control among individuals with hypertension [6]. The literature provides ample information on health education programs to support self-management for individuals with hypertension living in high-income countries [7]. However, information on the impact of the best possible education programs for individual with hypertension in low-resource countries is not available [8]. To fill the gap, in this study, we provide periodic health education aimed at increasing awareness on the relationship between BP level and salt intake, and knowledge about behavioral changes related to hypertension for positively influencing patients’ perceptions of hypertension and their adherence to treatment remedies [9].

However, the current status of the knowledge, attitudes, and perceptions of hypertension among participants indicates the significance of awareness buildup with respect to various features of hypertension, basic changes in lifestyle, and detailing of procedures in improving health education. Moreover, participants’ knowledge about hypertension and the benefits of lifestyle modifications by all means is important for successful management of hypertension [10]. Nevertheless, lifestyle changes are not effectively accomplished. Hence, a combination of health education, knowledge of behavioral changes, and periodic use of mobile health (mHealth) technology could be provided in a patient-centered manner [11], as adherence to treatment is likely to increase when participants have positive attitudes toward controlling hypertension. Subsequently, well-structured educational interventions along with active participation of the patients are vital to increasing the knowledge, self-monitoring, and control of hypertension.

To expand awareness of the target population, innovative techniques are introduced in this study. One is regular BP checkup by the use of a Portable Health Clinic system (developed by Kyushu University, Japan, and Grameen Communications, Bangladesh) during home visits by community health workers (CHWs), in light of the fact that a routine health checkup system at the community level is yet to be established in Bangladesh, particularly in its rural areas. For example, medical personnel such as community nurses, during their field visits, may routinely check the BP of hypertensive individuals, who do not have any BP self-checking facility at home.

The second extraordinary contraptions are used to measure food and urine salinity to alert the participants. The third strategy is the persuasive motivating approach and social capacity building using mHealth technology for behavioral change.

We hypothesize that awareness development and knowledge acquisition can enhance behavioral change, and periodical mobile phone–based SMS intervention will be significantly beneficial in modifying lifestyle changes among hypertensive individuals. Thus, this approach could minimize the hypertension status of participants as well as its complications.

### Study Objectives

The objectives of this study are to develop awareness and enhance knowledge through health education, the use of mHealth technology, and change in lifestyle behaviors among hypertensive individuals in a rural community of Bangladesh.

## Methods

### Design

This is a single-center, randomized (1:1), open-label, parallel-group study, conducted in a rural community of Mirzapur, Bangladesh. The intervention period was 5 months for each individual, and the total study duration was 12 months.

### Study Population and Sampling

Individuals with hypertension living in the study site were identified as follows. One group of individuals were identified from a tertiary-level health facility (Kumudini Hospital, Mirzapur, Bangladesh). The principal investigator and CHWs visited the tertiary facility and checked the database records as well as the registered notebook including current remedies from the facility and collected household contact information (ie, home address and phone number) with the permission of the hospital authorities. Afterwards, CHWs visited homes of the participants and obtained their consent to participate in this study. The other group of individuals were identified from the neighborhood rural communities arbitrarily by CHWs reviewing the registered clinician’s prescriptions and current remedies by household visits, and obtained their consent for the study.

A purposive sampling method was followed for enrolling study participants who met the eligibility criteria and were willing to participate voluntarily in the study.

Participants who met the following criteria were eligible for enrollment into the study:

Individuals of either sex who have hypertension and are aged 35 years or above, have 1-5 years of schooling, reside within a radius of 3 miles from the Kumudini Women’s Medical College and Hospital, are likely to stay in the community for the ensuing 5 months, have a personal cell phone or access to a shared phone, are open and can exchange their views freely, are willing to participate in the study, and consented to comply with the health education and periodic text messages for the entire study period.

Individuals with mental illnesses or serious comorbidities such as progressive diabetes, chronic pulmonary disease, malignancy, and pulmonary tuberculosis that might cause periodic absence were excluded from this study.

### Randomization

A randomization schedule was prepared following the permuted block randomization technique using a block size of 4 based on a computer-generated series of numbers. An experienced researcher (third party) who was not involved with the study generated the random allocation by sequence and distributed that in serially numbered sealed opaque envelopes, which are kept in locked file cabinets in a site away from the trial’s location. Sealed envelopes containing group assignment were given to the eight CHWs who were involved in participants’ enrollment under the guidance of a principal investigator.

Enrollment took place during morning hours for 6 consecutive days (except Friday) in a week, and the principal investigator of the study was present at the site every day to ensure compliance with the study protocol. Once eligibility for randomization was determined, written informed consent was obtained. CHWs opened the envelope in the presence of participants. Participants were allocated to either of the groups, after enrollment, CHWs collected their demographic information and performed physical examination of the participants.

### Study Procedure

#### For the Intervention Group

**Figure 1:**
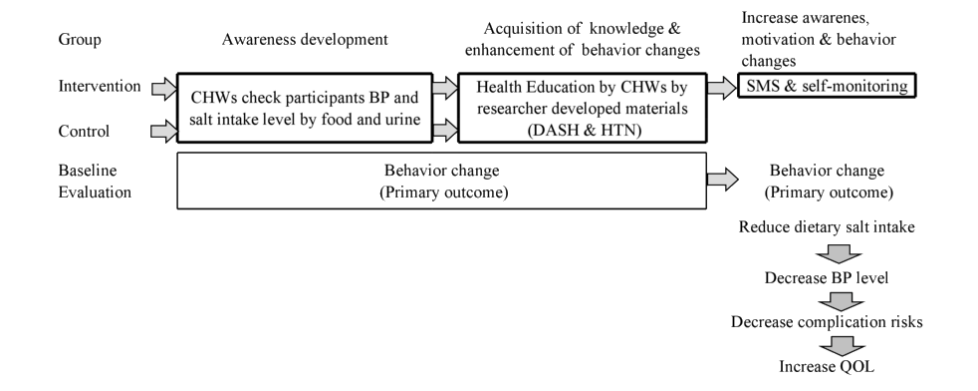
Awareness development process and contents of study outline.

To develop awareness, enhance knowledge, and motivate behavioral changes using mHealth technology, the intervention group received the 5-month health education including health education materials and SMSs (Figure 1). At the time of enrollment, CHWs visited participants’ households or asked participants to come to the nearest health facility such as Kumudini Women’s Medical College and Hospital or Health and Family Welfare Center (HFWC) in the morning. CHWs interviewed them; performed physical examination (measurement of BP, height, weight, mid-upper arm circumference, waist-hip circumference) of the participants; and measured random blood sugar for about 30 minutes. Along with these, CHWs checked food salinity by measuring salt from liquid foods and urinary salinity by spot urine and first morning urine. Along with this, they checked urinary glucose and protein levels for another 30 minutes. Subsequently, CHWs explained the data and provided health education by researcher-developed booklets based on the dietary approach to stop hypertension (DASH) diet and lifestyle-changing behaviors like salt intake reduction, smoking cessation, exercise, and medication (in Bangla or English; Multimedia Appendix 1). Participants of the intervention group were followed up every month up to 5 months (twice in the first month and once in the rest of the months). Text messages were sent 5 times in the first month and once a week for the remaining 4 months (a total of 16 text messages).

#### For the Control Group

The control group received the same health education booklet as the intervention group at the time of enrollment. After that, CHWs visited and provided health education at participants’ households or asked them to visit the nearest health facilities such as Kumudini Women’s Medical College and Hospital or HFWC. They were followed up every month for up to 5 months (twice in the first month and once the rest of the months) for receiving health education only.

### Sample Size

Sample size calculation was based on the behavioral changes of study participants. In this study, investigators compared two interventions (health education with and without text messaging). Investigators expected the compliance of the intervention group to be higher than that of the control group. We assumed that the proportion of patients in compliance is at most 10%-12% better in health education with text messaging group.

The sample size was calculated with a two-tailed 5% significance level and a power of 80%, with a 95% CI (1–α) to detect varying differences in the effectiveness of the two intervention groups. Adherence rates were assumed to differ by 10%-12%, that is, rates of 90% in the study group and 78%-80% in the control group, with a presumption that 6% of the participants would be lost during the follow-up (for example, if the intervention group adherence rate is 10% higher than that of the control group, then the difference will be 90%–80%=10%; if the intervention group adherence rate is 11% higher than that of the control group, then the difference will be 90%–79%=11%). The sample size for each group was estimated to be between 153 and 210 participants. Thus, considering the largest calculated sample size, the study finally had a sample size of 210 in each group.

### Outcome Measures

Once the participants were made aware of the importance of lifestyle changes and obtained knowledge about lifestyle modification, they could change their lifestyles. Thus, increased awareness and knowledge was measured by the actual behavior change in them. Following the lifestyle change, their BP level was expected to decrease, the risk for complications would be reduced, and their quality of life would be improved. Therefore, we set the following end points.

#### Primary End Point

The primary outcome was the evaluation of the behavioral changes using the researcher-developed questionnaire (Table 1).

**Table 1:** Questionnaire for the evaluation of behavioral changes.

Evaluation questions	5^a^	4^b^	3^c^	2^d^	1^e^
1. How many days do you eat fruits in a week?	Everyday	5-6 days/week	3-4 days/week	1-2 days/week	0 day/week
2. How many days do you eat vegetables in a week?	Everyday	5-6 days/week	3-4 days/week	1-2 days/week	0 day/week
3. How much salt do you take per day in a week?	42 g (<6 g/day)	5-6 days/week (7-8 g/day)	3-4 days/week (9-10 g/day)	1-2 days/week (11-12 g/day)	0 day/week (13-14 g/day)
4. How many days do you do 30-minutes physical activity/exercise in a week?	Everyday	5-6 days/week	3-4 days/week	1-2 days/week	0 day/week
5. How frequently do you check your blood pressure in a month?	8 times/month	6 times/month	4 times/month	2 times/month	Never/month
6. How frequently do you monitor your body weight in a month?	8 times/month	6 times/month	4 times/month	2 times/month	Never/month

^a^5: Excellent.

^b^4: Good.

^c^3: Fair.

^d^2: Poor.

^e^1: Very Poor.

#### Secondary End Points

Secondary outcomes were (1) the actual salt intake (measured by a salinity tester [TANITA electronic salinometer SO-313]) and dietary salt excretion (measured by KME-03, KOUNO ME Institute), (2) BP value, (3) blood glucose level (measured by EasyMate G, Model no. ET-111, Bioptik Technology Inc) and urinary protein and glucose (measured by urine test strip uric 2v Glucose Protein, Changchun Merydi Bio-Tech Co, Ltd) for checking complications, and (4) quality of life measured by EQ-5D-5L quality of life questionnaire [12].

Primary and secondary outcomes were measured every month including baseline and up to 5 months for both intervention and control groups.

### Feasibility Evaluation

As a secondary outcome, the feasibility of utilizing mobile phones in hypertensive individuals using field-testing questionnaires was checked by the researcher-developed questionnaire.

### Economic Evaluation

Researchers have strongly recommended economic evaluation of health promotion interventions such as health education and mHealth technology [13]. For this purpose, all costs were classified according to major activities or resources. Costs were derived from two phases of the intervention named start-up costs and implementation costs, which are directly related to the study. Total costs were considered as the summation of capital and recurrent cost items for each phase [14].

### Study Preparation

#### Training of Community Health Workers

CHWs were oriented on hypertension of rural community people every month till the completion of the study. Through this training, they understood the pathology and mechanisms of hypertension, dietary practices, behavioral changes, and physical activities that are needed for controlling hypertension. Moreover, they could perform physical measurements and acquire motivational skills that are useful for drug and instructional compliances by the rural community people and are essential for the prevention of complications.

#### Health Education and the Material/Notebook

Health education materials were developed based on the DASH diet (4 to 5 servings a day of vegetables and fruits, fat dairy foods, avoidance of excessive smoking and drinking), with advice to walk every day for at least 30 minutes and to take medicine regularly. DASH diet and suggestions to reduce hypertension are stop smoking and drinking, walk at least 30 minutes every day, and take medicines regularly [15-18].

#### Focus Group Discussion on Individual’s Hypertension Perception

An exploratory qualitative study was conducted to comprehend the knowledge level in relation to hypertension in a rural community of Bangladesh. The study assessed the level of understanding of individual’s perception regarding symptoms, causes, and consequences of hypertension, the importance of lifestyle modifications, and regular checkups. One of the critical aims was to investigate the practice of hypertension management, adherence to drugs, barriers and challenges that keep them away from seeking regular clinical consultation, laboratory investigation, and medication adherence.

#### Text Messaging Development and Testing

SMS text messages have been developed in native language by the members of the research team representing different professional backgrounds; these messages were sent to 15 people (randomly) for their comprehension and acceptability as part of the field-testing procedure. Individuals in the wired intervention group received SMS text messages, and they had the liberty to participate additionally in a two-way communication system (between intervention individuals and research team members). After receiving the feedback, messages were finalized. The SMS consisted of simple health education information to aid in the behavioral change of participants. Attempts were made so that all the participants in the study could read the SMS messages by themselves or by someone in the family who could read and explain the contents of messages to them if they do not understand. Contents of the text messages are presented in Table 2.

**Table 2:** Content of text messages. Text messages were scheduled to send 5 times for first month and once a week for the rest of the 4 months.

Message content	Examples
Reduce sodium intake (salt)	Sodium intake is < 6 g/day (just under a teaspoon). No extra salt, not even with fruits or green leafy vegetables, pickles, milk, or any other foods. It keeps your high blood pressure under control.
Avoid taking oily and fatty foods	Like beef, mutton, poultry, pastries, cakes, and other junk foods. You can gain weight.
Eat more fruits and vegetables	80 g per servings (fruits small size—full; large size—half; per meal) per day. It makes you healthy.
Do exercise regularly	Regular exercise (walking, running, cycling, household work) for 20-30 minutes most days of the week. It keeps your heart well.
Take your medicine regularly	Do not change and stop your hypertensive treatment without your doctor’s guidance.

### Measuring Predictor Variables for Intervention and Control Groups

At baseline, a standardized pretested questionnaire was administered to obtain information on the sociodemographic profile such as age; gender; religion; financial dependency; educational qualification; occupation; monthly family income and expense; type of housing; possessions of household land and properties; type of family; marital status of the respondent; living arrangements; health status; health care–seeking behavior; family history; hypertension-related information; hospitalization history; food consumption practices; lifestyle behavior such as tobacco use; alcohol consumption; food habits; the level of physical activity; and mHealth-relevant information.

Nutritional status assessments such as height, weight, mid-upper arm circumference, hip circumference, and waist circumference were measured by CHWs. BP, random blood sugar, urinary protein and glucose, and salinity of food and urine were measured using the standard procedure starting from the enrollment day till the end of the follow-up. Follow-up visits and physical examinations were performed at households, at Kumudini Hospital, or in the nearest HFWC.

### Ethical Consideration

All participants were explicitly informed about the objectives, importance, and risks and benefits of the research before recruitment. Participation was completely voluntary, and written informed consent was obtained from all participants.

This study was approved by the Bangladesh Medical Research Council (BMRC; Registration No. 06025072017), with Clinical Trial Registration No. NCT03614104 and UMIN Registration No. R000033736. This study was conducted in accordance with the Declaration of Helsinki and the Ethical Guidelines for Clinical Studies of the Ministry of Health, Labor and Welfare of Japan. Enrollment of participants started in August 2018, and collection of follow-up data was completed at the end of July 2019.

### Quality Control

In 5% of the study participants, the quality control team independently checked data collected on the same day using a field-tested methodology. Errors detected were corrected immediately at the field site. Later on, scoring was completed for each question of the repeated interview (eg, 1=same, 2=different but possible, 3=different and not possible, 4=impossible to judge, and 0=not applicable). The sum of all the scores was divided by the total number of questions/variables asked. The ideal score was 1 or a score as close to 1 as possible. If the score exceeded 1.5, the CHW responsible for the interview was questioned regarding the differences. The findings of the quality control team were considered for necessary corrections if any major discrepancies were found.

### Statistical Analysis

The intend-to-treat analysis was used to compare the outcomes of the intervention and control groups. To ensure comparability of randomized samples, all baseline indicators at the time of registration were analyzed. The data will be expressed as the mean±standard deviation or median (minimum-maximum) and cross tabulation (with the range or percentage with 95% CI where appropriate) for continuous variables and as frequencies and percentages for discrete variables. The differences in continuous variables between the groups were examined using the *t*-test or Mann-Whitney *U* test. The differences in categorical variables between groups were examined using χ^2^ test. For primary and secondary endpoint analyses, Mann-Whitney *U* test was performed to assess the changes in health behaviors at baseline, and analysis of covariate was performed to assess the changes in health behaviors at baseline and 5 months after the follow-up. Analysis of covariance was used to check the correlation between a dependent variable and the covariate independent variables and to remove the variability from the dependent variable that can be accounted for by the covariates. A multivariate regression analysis was then performed to evaluate the simultaneous effects of various exposure variables after adjusting for any confounding variables. Data were analyzed using the statistical software packages SPSS for Windows version 25.0 (IBM Corp) and Epi Info version 7.0 (Centers for Disease Control and Prevention). Values of *P*<.05 are considered statistically significant.

## Results

**Figure 2:**
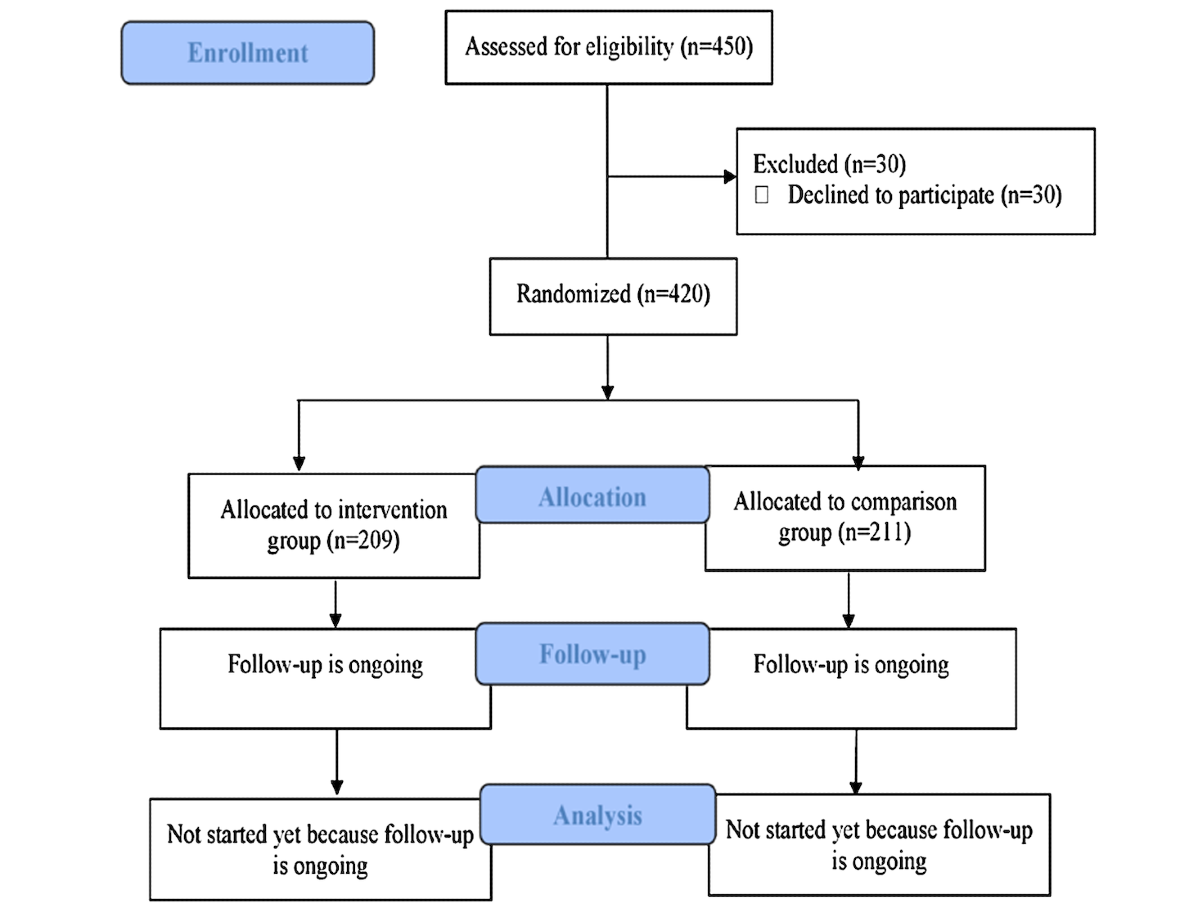
Enrollment procedure of the study.

Currently, the study is in the active implementation phase (participants’ follow-up is ongoing) after the completion of recruitment. A total of 420 participants have agreed to participate and among them, 209 and 211 are allocated to the intervention group and control group, respectively (Figure 2). The proportion of males/females is 12.0/88.0 in the intervention group and 16.1/83.9 in the control group.

From August 2018 to July 2019, the following processes were completed: recruitment, intervention, and data collection. Subsequently, data cleaning was completed, and from the results, data analysis (eg, of baseline sociodemographic data) was performed. Preliminary analytical results are presented in Table 3.

**Table 3:** Age-sex breakdown of study participants.

Variable		Intervention group (n=209), n (%)	Control group (n=211), n (%)
**Age group (years)**			
	35-44	84 (40.2)	70 (33.2)
	45-54	83 (39.7)	91 (43.1)
	55-64	34 (16.3)	39 (18.5)
	≥65	8 (3.8)	11 (5.2)
**Gender**			
	Male	25 (12.0)	34 (16.1)
	Female	184 (88.0)	177 (83.9)

## Discussion

### Principal Findings

This paper describes the protocol for a randomized control trial that aims to develop awareness, enhance knowledge, and make behavioral changes using health education and mHealth technology among individuals with hypertension in a rural community of Bangladesh. To our knowledge, this is the first study conducted in Bangladesh to measure the impacts of mobile phone SMS as a tool for reducing the burden of hypertension. One of the most significant barriers to effective treatment of hypertension is the lack of awareness and education about hypertension, its complications, and the optimal way to treat hypertension, which can be addressed by mobile phone interventions for knowledge generation as well as awareness and behavior change communications.

### Strengths

In this study, we sent SMSs to participants through mobile phones (two-way communication). As the SMS is written in *Bengali* using *Bangla* alphabets, we assume that it will not be very difficult for the participants to read and understand, as the ability to read the SMS messages has been considered in the inclusion criteria. Using SMS as a tool for health education, health information and lifestyle messages can be easily disseminated by persons with minimum technical knowledge and skills.

Moreover, in our study, we have used the following three unique devices to improve the self-management skills of individuals with hypertension.

#### Portable Health Clinic

Portable Health Clinic [19] is a handy, portable box that contains devices to measure BP, blood sugar, and urine protein and glucose and has an automatic transmission function to provide results (Multimedia Appendix 2). During their home visits, CHWs measure the parameters, and after checking that the data are fed immediately and used for health education.

#### Urine Salinometer

This is a handy, instant measurement device that can estimate the participant’s salt intake of the previous day at home (Multimedia Appendix 3). Participants’ baseline urine is analyzed as a proxy for daily salt intake and follow-up urine in the morning is analyzed using a KME 03 salinometer (developed by KOUNO ME Institute) [20].

#### Food Salinometer

We also check the participants’ food salinity as a proxy for their daily salt intake by using an electronic salinometer for measuring food (TANITA white waterproof salinometer SO-313, which has three different types of level sensors; Multimedia Appendix 4). It starts from level 0.4 to 0.7 (yellow color), 0.8 to 1.1 (green color), and 1.2-1.4 (red color). It is a three-level salinity tester for food items. A range of 0.4-1.4 g of salt in the food can be measured using this device.

### Limitations

Because of purposive sampling, this study does not represent hypertensive individuals of a rural community as a whole. To check the feasibility and reliability of urine and food salinity results, it is better to compare them with laboratory data, which cannot be evaluated in this study. CHWs were consented not to share the random allocation status of hypertensive individuals with the family members as well as their neighbors during the study period. However, chances of disclosure, although small, cannot be ruled out. Moreover, data contamination due to neighborhood as well as family members and obtaining consent from CHWs to not disclose the data may be other limitations of the study.

### Conclusions

This study was conducted in rural Mirzapur, Bangladesh. This study aimed to determine the effectiveness of SMS-based interventions for health promotion and to determine whether it will be well accepted by beneficiaries. The results of the study indicated that SMS is an effective method for building awareness toward the prevention and control of hypertension and its consequences in a rural population of Bangladesh [21-23].

## Appendix

Multimedia Appendix 1 Health education material.

Multimedia Appendix 2 Portable health clinic device.

Multimedia Appendix 3 Urinary salinity check by salinometer.

Multimedia Appendix 4 Food salinity check by food salinometer.

## Abbreviations

BP: blood pressure
CHWs: community health workers
CVDs: cardiovascular diseases
DASH: Dietary Approach to Stop Hypertension
HFWC: Health and Family Welfare Centers
mHealth: mobile health
