# Supplementary figures and images for: Increasing Awareness and Use of Mobile Health Technology Among Individuals With Hypertension in a Rural Community of Bangladesh: Protocol for a Randomized Controlled Trial
Source: JMIR Res Protoc. 2020 Aug 17;9(8):e15523. doi: 10.2196/15523 (PMC7459433; doi:10.2196/15523)

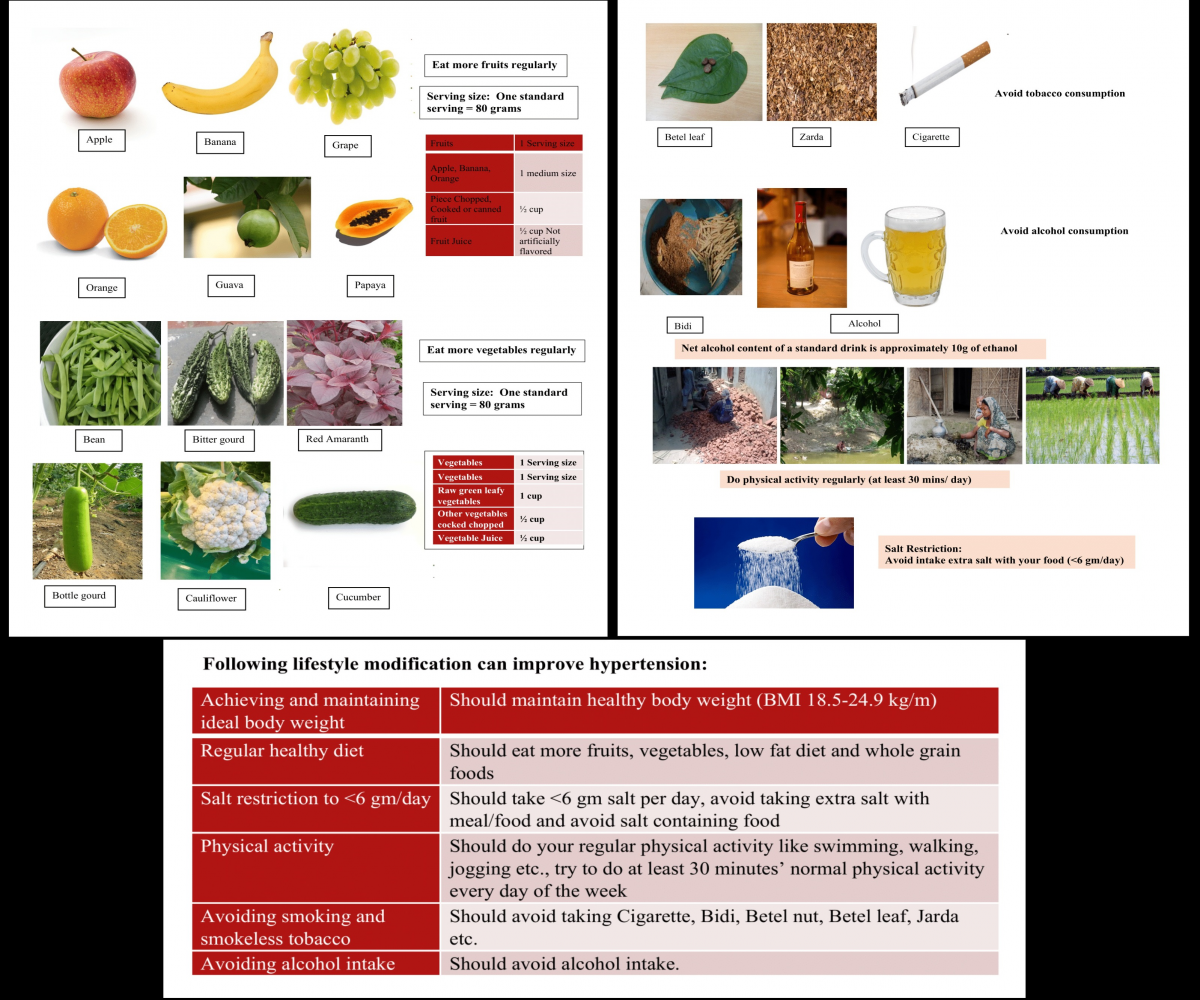

Supplement: Multimedia Appendix 1 [file resprot_v9i8e15523_app1.png]

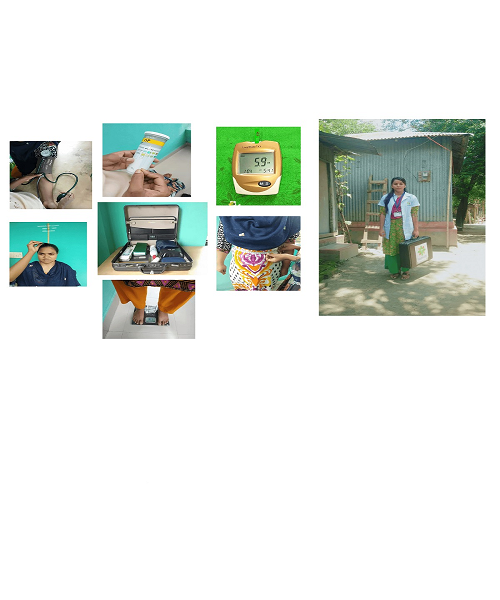

Supplement: Multimedia Appendix 2 [file resprot_v9i8e15523_app2.png]

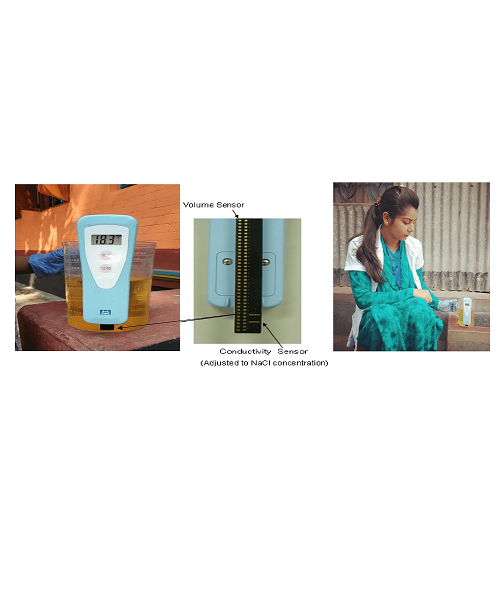

Supplement: Multimedia Appendix 3 [file resprot_v9i8e15523_app3.png]

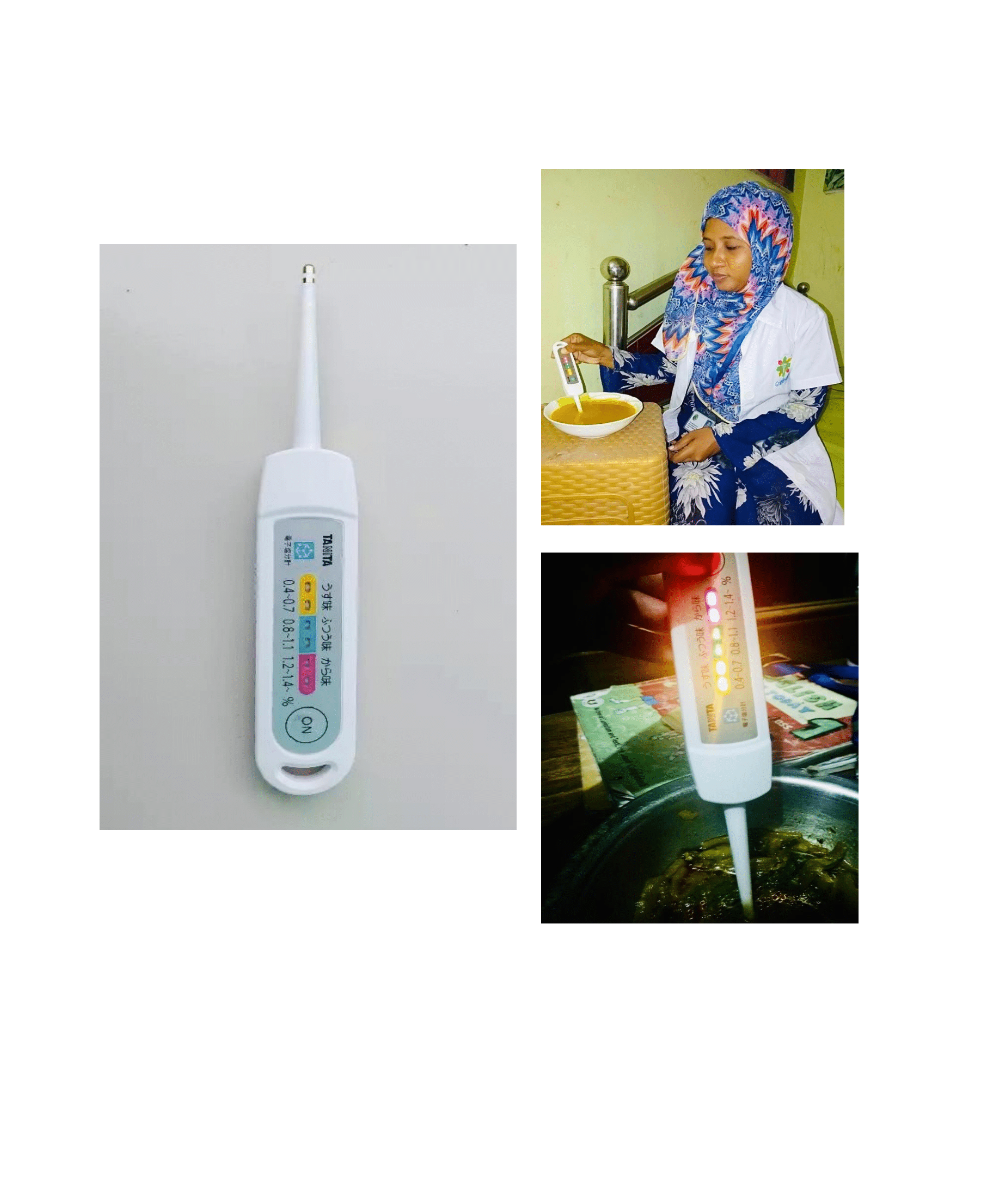

Supplement: Multimedia Appendix 4 [file resprot_v9i8e15523_app4.png]
